# *“What if I get ill?”* Perinatal concerns and preparations in primi- and multiparous women with bipolar disorder

**DOI:** 10.1186/s40345-019-0143-2

**Published:** 2019-03-03

**Authors:** Teija M. S. Anke, Kari Slinning, Dag Vegard Skjelstad

**Affiliations:** 1 Division of Mental Health and Addiction, Vestre Viken Hospital Trust, 3004 Drammen, Norway; 2 Centre for Child and Adolescent Mental Health Eastern and Southern Norway, R.BUP, Oslo, Norway; 3 Vestre Viken Hospital Trust, Drammen; University of Oslo, Oslo, Norway

**Keywords:** Bipolar disorder, Perinatal, Postpartum, Primiparous, Multiparous, Mood episodes, Concerns, Preparations, Prevention, Counselling

## Abstract

**Background:**

Women with bipolar disorder have a high risk of illness relapse postpartum, including psychosis. The aim of the study was to explore how perinatal women with bipolar disorder relate to the risk. What are their concerns? How do they prepare for the dual demands of mood episodes and motherhood?

**Methods:**

A qualitative study was conducted. To ensure rich insight into the research questions, 13 primiparous and 13 multiparous women with bipolar disorder (I or II), were individually interviewed in pregnancy or early postpartum. Thematic analysis was applied.

**Results:**

Across parity, concerns for illness relapse included concerns for depression and psychosis. Primiparous women worried about “the unknown” in relation to postpartum reactions. Overall, the most significant concerns were the impact of mood episodes on mothering and on the partner. Concerns regarding the infant were maternal medication, mood episodes affecting the child, and heredity. Resources and preparations included: support from the partner, the family, and health services; adjustment of daily life; and mental strategies. Women were aware of the postpartum risk, but their levels of personal concern varied between low, moderate and high. Women with low level of concern for illness relapse had made the least deliberations and preparations. A subgroup of women with high level of concern also had limited resources and preparations.

**Conclusions:**

The findings highlight the importance of including a psychological and psychosocial focus in perinatal prevention planning and counselling. Even if women with BD are informed about the increased risk of illness relapse postpartum, they relate to it differently. Their level of personal concern impacts their perinatal deliberations and preparations, which in turn may impact postpartum adjustment. When counselling these women, it is important to assess their personal risk recognition, perinatal concerns and available resources and preparations, and support them accordingly. Extra attention should be given to women with a low level of concern, and women with a high level of concern who have limited resources and preparations. These women represent particularly vulnerable subgroups that are critical to identify and offer comprehensive follow-up.

## Background

For women with bipolar disorder (BD), childbirth and the postpartum period are associated with significantly heightened risk of illness relapse (Pope et al. 2014). A recent meta-analysis found an overall relapse risk of 37% within the first year postpartum and most relapses occurred within 6 months. Relapse was less common among women on prophylactic medication during pregnancy (23% vs. 66%). For 17% the relapse was severe, requiring hospitalisation (Wesseloo et al. 2016). Despite the high prevalence of postpartum episodes, there is a scarcity of studies regarding perinatal management of BD (Jones et al. 2014; Pope et al. 2014; Yatham et al. 2018).

Furthermore, existing literature is mainly related to illness course and medication (Sharma and Sharma 2017; Yatham et al. 2018). These are indeed crucial issues, but managing BD in the perinatal period is complex and involves challenges that exceed pure medicational matters (Jones et al. 2014; Pope et al. 2014). Moreover, postpartum mood episodes occur in a psychologically sensitive phase of life, with significant implications for the mother, the infant and the father (Sutter-Dallay et al. 2016; Tyano et al. 2010). Hence, a growing body of literature recommends a more comprehensive follow-up which includes psychological and psychosocial matters (Howard et al. 2014; Meltzer-Brody and Jones 2015; Pope et al. 2014; Sharma and Sharma 2017). However, there is a significant knowledge gap regarding the perceived needs of women with BD in relation to pregnancy, childbirth and motherhood (Rusner et al. 2016).

To our knowledge, there are only two previous studies focusing on perinatal matters for women with BD from an experiential viewpoint (Dolman et al. 2016; Stevens et al. 2017). Both studies addressed family planning and pregnancy, and the women’s decision-making on whether to become pregnant or not. The decision-making process was complex, and many women did not feel appropriately supported by health professionals (Dolman et al. 2016; Stevens et al. 2017).

To learn more about the concerns and preparations of perinatal women with BD, the present study aimed at exploring how women with BD relate to the increased risk of illness relapse postpartum: How do they perceive the risk? What are their concerns regarding the possible impact of BD for mothering and their families? What resources do they make use of in preparing for the dual demands of illness and motherhood? To ensure rich insight into these research questions, a group of primiparous (PP) and multiparous (MP) women with BD diagnosis was interviewed. The overarching aim was to gain knowledge that may inform prevention planning and counselling for perinatal women with BD.

## Methods

### Design

The study is part of a Norwegian prospective investigation on families in which the mother has BD. Data were collected at four time points from pregnancy to 12 months postpartum. For the present study, a qualitative approach was chosen to explore subjective experiences in an open and inclusive manner. We used interviews to gain detailed and rich information.

### Recruitment procedures and participants

The study included 26 pregnant or postpartum women with a BD I or II diagnosis. Because of the aims of the larger investigation they had to have a cohabitating partner who was willing to participate. Exclusion criteria were parental substance abuse; multi childbirth; premature birth < 35 weeks; or having an infant with a known serious medical condition or syndrome. These parental and child conditions could bias the interaction observations that are included in the larger investigation.

Recruitment took place between September 2014 and July 2016. Most participants (n = 15, 58%) were recruited from the geographic area of Vestre Viken Hospital Trust in Norway, with a population of 490,000. The remaining participants were recruited from nearby counties in the South-Eastern part of Norway. We provided oral and written information about the study at mental health outpatient clinics and wards, infant mental health teams at child mental health services, community well-baby clinics, pregnancy care and maternity wards. The health professionals then distributed information leaflets and asked eligible women whether a clinical psychologist and researcher (T.A., first author) could contact them by phone to provide more details about the study.

A few participants were recruited from the web-site of the national BD association and at group psychoeducation courses for patients with BD (Skjelstad et al. 2015).

Women and partners who were willing to participate gave oral and written consent at an information meeting with T.A. The consent allowed T.A. to have the women’s clinical BD diagnosis verified by examining their specialist mental health patient records and/or by contacting their specialist mental health worker. All participants had received their diagnosis in the specialist mental health service. Further details concerning the history, presentation, and treatment of the BD were covered in the study interview. It was deemed unnecessary to reassess any BD diagnosis with a semi-structured diagnostic instrument.

Thirty-five women were interested in more information about the study. In three cases, the woman’s partner declined to participate. Three women turned out not to have a formal BD diagnosis. One woman did not respond to T.A.’s calls for setting up a meeting and two women changed their mind. Thus, the final sample comprised 26 women diagnosed with BD (13 PP and 13 MP). We have no record of the number of eligible women who declined to participate when informed by collaborating health professionals.

### Data collection

Nineteen women (73%; 8 PP and 11 MP) were interviewed in pregnancy (mean 32 weeks, range 21–39), and seven (27%; 5 PP and 2 MP) in the early postpartum period (mean 11 weeks, range 4–16). The interviews were conducted by T.A., either at the participant’s home (77%) or at T.A.’s office, as chosen by each woman.

In the interview, the women were asked to describe their experiences of the pregnancy, their thoughts on becoming a mother with BD, and how they envisioned managing the dual demands of illness and motherhood. MP women were allowed to refer to previous pregnancies and postpartum periods when they felt it relevant.

An interview guide was used to ensure that the topics were covered across all interviews. The order in which questions were presented was adapted to the natural development of each interview. First, the questions were posed in an open-ended manner to invite in-depth descriptions. Probe questions were then used to encourage elaborations. The interview guide also contained some questions with fixed responses, including a question about satisfaction with life conditions. These topics were also elaborated on.

The interviews lasted approximately 1 h (mean 70 min, range 35–109). Audio was recorded and transcribed.

### Data analysis

An inductive thematic analysis was applied, because of its’ suitability for investigating understudied topics (Braun and Clarke 2006; Braun and Clarke 2013). Each interview was listened to and read repeatedly by the first author. With the research questions in mind, initial codes were generated case by case (Braun and Clarke 2013, p. 206). The codes reflected salient features in text segments. The next step was to look for patterns and similarities among the different codes in order to organise them into candidate themes. Codes and candidate themes were then modified in a flexible “dialogue” with the data (Braun and Clarke 2013, p. 224).

The third author read all the transcripts and supervised the analysis. Interpretations and candidate themes were discussed throughout the analytic process and revised until agreement was reached. Distinct themes were named and organised hierarchically in collaboration. The second author, who has extensive experience in perinatal mental health, addressed possible omissions and contradictions in the analytic process. Interpretations were also discussed in peer groups. Finally, the material was analysed across the cases with the purpose of identifying similarities and differences between PP and MP women.

An interviewee validation was done at a second interview at 12 months postpartum. The participants were presented with verbal summaries of their first interviews. All participants confirmed that their viewpoints were portrayed accurately.

The qualitative software NVivo 10 was used as a coding and organising tool.

## Results

### Demographic and clinical characteristics

Table 1 shows the demographic characteristics of the sample. All women lived with the infants’ biological father. In the MP group, 85% had their second pregnancy.

**Table 1:** Demographic characteristics of the sample

Variables	Total N = 26		Primiparous N = 13		Multiparous N = 13	
Age at inclusion	M 30.5; range 22–37		M 28; range 22–36		M 32.9; range 25–37	
Satisfaction with life conditions^a^	M 4.2; range 3–5		M 4.2; range 3–5		M 4.2; range 3–5	

	n	%	n	%	n	%
Planned pregnancy	16	62	5	38	11	85
Completed education						
Primary school	8	31	5	38	3	23
Secondary school	5	19	2	15	3	23
Bachelor’s degree	11	42	6	46	5	38
Master’s degree	2	8	0		2	15
Employment status when not pregnant						
Working full-time	12	46	6	46	6	46
Working part-time ± receiving benefits	4	15	2	15	2	15
Receiving benefits only	8	31	3	23	5	38
Unemployed	1	4	1	8	0	
School	1	4	1	8	0	

^a^Question in interview: “How satisfied are you with your current life conditions—regarding housing, economy and such?” Answers on a 5 point Likert scale: 1 = dissatisfied, 2 = not especially satisfied, 3 = moderately satisfied, 4 = satisfied, 5 = very satisfied

Table 2 displays clinical characteristics of the sample. The majority (73%) had mental health struggles, including affective problems, since before age 16. We do not report comorbidity diagnoses because of incomplete data.

**Table 2:** Clinical characteristics of the sample and the health status of partners

Variables	Total N = 26		Primiparous N = 13		Multiparous N = 13	
Years with BD diagnosis^a^	M 6.5; range 0–16		M 5.2; range 0–16		M 7.8; range 2–13	

	n	%	n	%	n	%
Primary diagnosis						
BD I	7	27	4	31	3	23
BD II	19	73	9	69	10	77
Number of hospitalisations for BD episodes						
0	10	38	6	46	4	31
1–3	10	38	3	23	7	54
≥ 4	6	23	4	31	2	15
Perceived mood stability						
> 1 year prior to pregnancy	11	42	4	31	7	54
Medicated for BD during pregnancy	17	65	7	54	10	77
Specialist mental health service in pregnancy						
Adult mental health	14	54	5	38	9	69
Infant mental health^b^	4	15	3	23	1	8
Both adult and infant mental health	2	8	2	15	0	
None	6	23	3	23	3	23
Health status partner (N = 26)^c^						
Mental problems	5	19	4	31	1	8
Medical problems	1	4			1	8

^a^Self-reported in interview: “When did you get your diagnosis?”

^b^Mental health service for prevention of mother-infant relationship difficulties

^c^Self-reported by partners as part of the larger investigation

### Thematic results

The thematic analysis generated two superordinate themes, named as “Concerns” and “Resources and preparations”. Within “Concerns” we identified four themes: “Illness relapse”, “Early mothering and mood episodes”, “Perinatal impact on child” and “Illness impact on partner”, with corresponding subthemes. Within the superordinate theme “Resources and preparations”, we identified the two themes “Supportive network” and “Personal strategies”, with related subthemes.

In the analysis we recognised that women expressed different levels of concern for a postpartum illness relapse. The levels of concern are detailed in the beginning of the theme “Illness relapse”. Correspondingly, we recognised different distributions of resources and preparations among the women. These are described in the beginning of the superordinate theme “Resources and preparations”.

### Concerns

Table 3 shows the four themes and subthemes of concerns with illustrative quotes, and the number of primiparous (n = 13) and multiparous (n = 13) women addressing the themes.

**Table 3:** Themes and subthemes of perinatal concerns

Illness relapse	Early mothering and mood episodes	Perinatal impact on child	Illness impact on partner
*The unknown*		*Medication*	
*“I am concerned about how my body will react after giving birth, how things will work hormonally and all that. We don’t know, even if all is well now.”* (P17, PP) 7 PP	*“It’s about the attention. When you are depressed, you don’t have the energy. When you are hypomanic, you don’t have the time. So it impacts you as a mother in both states.”* (P12, MP) 9 PP; 8 MP	*“I have been afraid that there will be something wrong with the baby, because of the medication, especially since I take so many different kinds of medications.”* (P10, MP) 4 PP; 4 MP	*“He is very good at not letting the depression affect him so much. He manages to see the positive in things and tries to have me do the same. But the postpartum psychosis I had, that was heavy for him.”* (P2, MP) 7 PP; 10 MP
*Depression*		*Heredity*	
*“If I have a bad day, I get a little panicked, because I’m so afraid that the bad day will stay there for a long time. What if this is the start of a depression?”* (P23, PP) 6 PP; 6 MP		*“I hope it’s the father’s genes that are passed on. I think a lot about it, and hope that he won’t become ill and unstable, like me.”* (P11, MP) 4 PP; 4 MP	
*Psychosis*		*Mood episodes affecting foetus or infant*	
*“I had psychosis after my last birth. So, of course there is this fear that it will come back again (…) It’s frightening to think that I have it in me.”* (P2, MP) 4 PP; 2 MP		*“I have been afraid that I won’t be patient enough, and if I have a “down trip”, will he notice?”* (P21, PP) 8 PP; 1 MP	

## Illness relapse

First, we describe “how” the women related to the risk for illness relapse, with different levels of concern. Then, we describe the content of the concerns.

### Levels of concern for illness relapse

The women were aware of the postpartum risk of illness relapse, but it varied whether they related to this risk personally and with concern. Overall, 73% of the women expressed explicit concern for illness relapse, whereas the remaining women had vague or rejecting declarations.

To further explore the levels of concern and the characteristics of women, we organised the women into three subgroups according to their perceived concern for postpartum illness relapse: low level of concern (LC: n = 7; 3 PP, 4 MP; 1 BD I, 6 BD II), moderate level of concern (MC: n = 9; 2 PP, 7 MP; 3 BD I, 6 BD II) and high level of concern (HC: n = 10; 8 PP, 2 MP; 3 BD I, 7 BD II).

The LC group described few thoughts about the risk—*“I don’t foresee any mood shifts. It’s not something I think about.”* (P22, PP)—or referred to previous postpartum experiences, *“All went well last time, so I think it will go well this time too, most likely.”* (P11, MP).

The women in the MC group explicitly acknowledged being at risk, but had it mostly in the back of their minds—“*I know that it’s a very vulnerable and difficult phase, the first 2* *months…but I am not that worried, because I have decided to take Lithium after birth.”* (P3, MP).

HC women were more emotionally activated in the interviews, expressed recurrent and elaborate descriptions of becoming ill, with an accompanying use of strong emotional words, such as “fear”, “frightening”, and even “horror”. Some women revealed having had marked concerns long before pregnancy—“*This horror of becoming ill (…) has actually ridden me for many years in deciding whether to have a child or not.”* (P17, PP).

Most (71%) women with LC perceived themselves as currently “stable” (euthymic). The women were often younger, had a lower level of education and work participation, had fewer planned pregnancies, and reported longlasting relational conflicts with own background family.

The majority (78%) of women with MC had completed secondary school or a bachelor’s degree, worked full time, were euthymic for at least 1 year prior to pregnancy, and had planned pregnancies. Many (78%) had close relatives with BD diagnosis.

Eighty percent of the HC women reported mood instability prior to and during pregnancy. Over half of the women (60%) had previously experienced severe illness episodes, and had the highest life time prevalence of hospitalisations. They varied regarding education, work participation and how long they had had their BD diagnosis.

The proportion of women being on medication was similar across groups. The number of women within each group being interviewed in pregnancy vs. postpartum were: LC 6 vs.1, MC 5 vs. 4, HC 7 vs. 2.

### The unknown

Over half of the PP women (54%) were worried about the unknown—not having any experience in how their illness may be affected by childbirth and the hormonal changes. *“There is this fear, where there should have been joy. You know, having a risk pregnancy.”* (P19, PP). None of the MP women reported such worries.

### Depression

The most frequent concern related to illness relapse was depression (46%), evenly expressed by PP and MP women. The women reported being vigilant of depressive symptoms in pregnancy. On the one hand, this could be confusing and stressing. On the other hand, it could be valuable for prompt interventions—*“I recognised that it was going downwards. The hopelessness came, the negative thoughts came, and I doubted whether I could manage. I imagined a postpartum depression, so I just had to stop it all (i.e. work, engagements).”* (P18, PP).

### Psychosis

A concern for postpartum psychosis was evident in five women with BD I (71%), and in one woman with BD II (5%), thereby being more related to type of BD than parity. Psychotic episodes with experiences of not being in control of oneself, having a fear of doing harmful things, and being judged by others as “mad”, were worrisome to think of in pregnancy—*“I become very strange when I am ill. People around me say that it’s not “me”. I become like another person, say strange things, and do strange things.”* (P15, PP).

A PP woman (P17) was concerned about the nature of the treatment she would be subjected to, should she experience a postpartum psychosis. Will it differ from previous interventions? Where would she be hospitalised? Would it be by force? Even though she was familiar with hospitalisation from several previous psychoses, the thought of it felt different and strange in pregnancy.

## Early mothering and mood episodes

A baseline perspective among the women was that given euthymia, BD has no impact on their mothering. Another common view was that the impact depends on the intensity of mood deviations. Mild intensity was viewed as rather unproblematic, as “healthy mothers experience it too”. Nevertheless, 65% of the women expressed concern for mood episodes affecting them as mothers.

Being naïve to mothering, PP women were mainly concerned from a hypothetical stance—*“What, then, if I get a huge “down trip” and I am supposed to take care of her? What kind of mother will I be then if I don’t function?”* (P26, PP). Some also feared that a postpartum relapse could affect maternal affection and bonding—*“That is something I’m very afraid of, that I might not want my child. That would be terrible.”* (P18, PP).

In remembering prior pregnancies, MP women validated doubts concerning own mothering in depressive episodes—*“When I was depressed at the end of pregnancy last time, I didn’t believe I could manage to be a mother. I just couldn’t understand how I could be able to be a mother.”* (P2).

In their current pregnancy, MP women’s concerns were based on lived experiences of mood episodes interfering mothering. Not being available, was a particularly salient concern, as in depression triggering withdrawal behaviour—*“I just need to be alone, and that weighs heavily on my conscience, because I have chosen to have a husband and a child. And still, I just lie here and don’t want to be with them.”* (P4, MP). Parallel to the PP women’s hypothetical concerns, a few MP women worried that the choice of an additional child might be pushing their limits too far.

Despite the concerns about mood episodes affecting their mothering, only a very small minority of the total sample (8%, 2 PP) explicitly expressed fear of child protection services—*“What if I get really psychotic for a long time? What if I get really ill and if our marriage breaks down? Then they will choose my husband as the caregiver. Why would they choose me?”* (P17, PP).

## Perinatal impact on child

Eighty-five percent of the women expressed at least one of the concerns related to perinatal impact on child.

### Medication

Sixty-five percent of the women were on medication (see Table 2). Approximately a third of the total sample, with even numbers of PP and MP, expressed concerns for how medication may affect the health of the foetus and the ability to breastfeed the infant. Women were more concerned about Lithium and antipsychotics, than Lamotrigine and SSRI medication. Additionally, some expressed concern for high doses and medication combinations.

### Heredity

An equal distribution of PP and MP women (31%), brought up concerns about heredity. Some thought that a stable and safe childhood could counteract the genetic risk—*“Well, the father is also bipolar, so of course I’m worried*. *But I also think that the circumstances the children grow up with matter…I mean, if a bipolar disorder will develop or not.”* (P7, MP).

### Mood episodes affecting foetus or infant

Particularly PP women (62% PP vs. 8% MP) were concerned whether an infant will notice mood shifts and be affected by them. This was often intertwined with the concerns for mood episodes affecting mothering, as in the case of impaired bonding and reduced emotional availability. Some women stressed the importance of being extra attentive with infants—*“It’s extra important to be aware of it with an infant, because they can’t speak up. They can’t come to you and say, “Mummy, why are you so strange?”* (P20, PP) Fussiness, crying and being tense were mentioned as possible infant reactions. Deviating from the PP women’s narratives, MP women primarily elaborated on consequences of mood episodes for toddlers or older children.

A minority (15%) worried that the foetus may be affected by their stress during mood episodes. This led them to find strategies to calm down in pregnancy.

## Illness impact on partner

All partners knew about their female partner’s BD diagnosis, but lived experiences with mood episodes varied. In the MP group, all partners had had such experiences, and 54% had experienced severe episodes needing hospitalisation. In the PP group, 77% of the partners had had experiences with the woman’s mood episodes, and 38% with severe episodes.

Both PP and MP women (54% PP vs. 77% MP) addressed concerns about the double strain impacting their partner: being affected by their mood episodes, while at the same time having to provide support (elaborated below in resources and preparations). Furthermore, several women described their partner as a counterweight to their mood instability in being calm and patient—*“He is very stable mentally. I think that’s important in a family in which the other isn’t. So I think that as a family we are doing well, but it’s obvious that most of the hard work falls on him.”* (P12, MP).

Some women reflected on the possibility that the partner keeps BD worries to himself—*“He seems very calm about all these things* (i.e. medication, illness relapse), *but it’s not unlikely that he keeps some things to himself, because he wants to support and normalise. But surely, he has his thoughts about things that worry him.”* (P19, PP).

A minority of PP women worried whether their partner would cope with a postpartum illness relapse—*“What worries me the most is how he will react and handle it, and how in turn I will handle him.”* (P16, PP).

### Resources and preparations

Table 4 shows themes and subthemes of resources and preparations, with illustrative quotes, and number of primiparous (n = 13) and multiparous (n = 13) women addressing deliberations about the themes.

**Table 4:** Themes and subthemes of perinatal resources and preparations

Supportive network	Personal strategies
*Partner*	*Adjustment of daily life*
*“He is a big, big support for me.”* (P2, MP) 7 PP; 8 MP	*“We are going to bottle feed the baby, partly because of my medication, but mostly because we feel it’s very reassuring that he (partner) can assist more and I can sleep.”* (P5, MP) 9 PP; 10 MP
*Family*	*Mental strategies*
*“My mother will take a week vacation after birth, so she can assist us. With regard to what happened to me last time (i.e. postpartum psychosis) she has decided to do that, and we appreciate it.”* (P2, MP) 10 PP;7 MP	*“We think it will work out this time, because now we know better what is ahead of us. We know a lot more about my illness and how it develops. I recognise the signs much earlier and take them more seriously than I did last time. Then I just thought, “Oh, this will work out well.” And the stuff about risk for psychosis afterwards…”No, that’s not about me.””* (P2, MP) 10 PP; 13 MP
*Health services*	
*“I think it is very important to talk about medications with the one who has the knowledge, not indirectly through my GP. If I have critical questions, then the GP has to contact the psychiatrist and it gets so much more complicated.”* (P2, MP) 9 PP; 8 MP	

### Distribution of resources and preparations among subgroups

The MP women tended to have more resources and preparations than the PP women, but there were variations. Overall, LC women (n = 7) had made the least deliberations and preparations. However, none were without resources or preparations. Among the MC women (n = 9), five of the MP women (n = 7) and one of the PP (n = 2) had sufficient access to resources and coping strategies. Among the women with high level of concern (n = 10) for illness relapse, six women, including both MP women, were extensively prepared. The remaining women (n = 4) had more limited preparations and resources.

## Supportive network

### Partner

The partner was described as an immense support by more than half (58%) of the women. Based on experiences, MP women described different kinds of support. A recurrent theme was the partner’s readiness to take night shifts with the infant, his active participation in child care and domestic chores—“*You know last pregnancy, I went all crazy, I got depressed and I panicked. I think he was shocked about how bad it really can get. But he handles it so well. He supports me 100%. He arranges everything, there’s really no end to what he does, because I need to rest a lot, even when I am not pregnant. He does a lot of the housekeeping, and doesn’t really complain, even if I say that it’s bad that it has to be like that. He says, “But, that’s how we function. Then our daily life works.””* (P5, MP).

Moreover, the MP women underscored the importance of the couple having a shared responsibility in taking the illness into account—*“We talk about it a lot, we do, and he says, “When you feel, if you feel, that it’s sneaking up on you, you feel that something is happening, then you have to tell.” And I do. So I think we manage quite well.”* (P10, MP).

PP women referred to the partner’s assistance in pregnancy and him being a safe and stable person, when expressing confidence in his postpartum support—*“I have felt it now in the pregnancy, how he arranges and fixes things*—*really “nesting”*—*he cares. He is present, and it seems like he has reflected a lot.”* (P18, PP).

Still, 23% of the PP women and 8% of the MP women explicitly expressed doubts about support from the partner. These women were either concerned about their partner becoming too affected by an illness relapse or that his own health problems might occupy his capacity—*“He is very ill, so it’s also very important for him to rest and sleep. As it is now, his health is a priority over mine. I have to function, but I think I go in “minus”, because there is so little support from him and I get tired.”* (P13, MP).

### Family

Several women (65%) displayed good hopes for family support when needed. Among MP women, 23% described that close access to family support had been a prerequisite in their planning to have their first child, whereas this held for 15% of the PP women—*“My parents are close all the way. They live nearby, and it’s both for the sake of me and our child, and not the least for the sake of my husband. Because when I get ill, he goes to them and they make plans together, and I don’t have to think about it. I don’t know if I would have been as relaxed about having children if we hadn’t had them. I think I would have been more reluctant. It definitely makes it easier. We talked with them before we decided to have our first child.”* (P4, MP).

A lack of family support was frequently conveyed with sadness (46% MP vs. 23% PP). Relational conflicts and/or the family living far away were common reasons—*“I have always had trust in my family, but I have lost it. And now,* (i.e. postpartum) *it’s an extra burden that I don’t have that safe setting anymore.”* (P24, PP) For some, a lack of family support was compensated by assisting friends—*“You do have people that are your, not your blood family, but they are your friend family. You know, you grow your friends. We are fortunate to have a lot of good friends.”* (P3, MP).

### Health services

Sixty-five percent communicated the importance of follow-up from specialist mental health services. Women expressed satisfaction when issues specifically related to the perinatal period and becoming a mother were addressed—*“I talked with a psychiatrist at the specialist mental health clinic. He is specialised in BD, and we talked about my pregnancy and the illness. One thing was the medication; can I use the medication I am on? Another thing was that everybody can actually have a postpartum depression. Things can happen in everybody’s lives, and some are physically ill. So there can be many reasons to you bringing on specific genes and not functioning all the time. You know, it doesn’t have to be that I become “the worst mum in the world” because of my bipolar luggage. I have reflected and worked a lot with myself. It may also be an asset*—*that you are self*-*conscious. So it was good talking with him.”* (P18, PP).

It was vital for the women to receive qualified guidance regarding perinatal medication, including effects on the foetus and advice on their decisions about breastfeeding. In these matters, several regarded the counselling in primary health care as unsatisfactory and wanted guidance from a psychiatrist in specialist mental health services.

Even numbers of PP and MP, (31%), had developed a specific birth plan in cooperation with health professionals. This included the possibility for a single room at the postpartum ward (to make the presence of the partner possible and to secure sleep), medications, and an option to prolong the stay in the postpartum ward. A couple of women had also given written consent to allow their partner to decide on forced hospitalisation in case of a serious illness relapse.

## Personal strategies

### Adjustment of daily life

Deliberations regarding daily life adjustments was mentioned by 73% of the sample. The need to secure sleep postpartum, was declared by 38% of the MP women, and 46% of the PP women. For 23% of the MP women the issue of securing sleep was decisive in deciding not to breastfeed. Besides sleep, several also mentioned the importance of rest and avoiding stress. The daily routines that come with having infants and small children were appreciated by several women—*“What’s been good with having a child is that there are routines. The routines, calming down and having less social life, those things have probably helped me to stabilise.”* (P4, MP).

### Mental strategies

We identified mental strategies among 88% of the women. A common strategy was consciousness about priorities. Mothering and “economising” with own energy, were described as two interconnected main priorities—*“There are things that have to be done, but it’s important that I don’t use all my energy dealing with them. The most important, is to use my time with my child, and with us as a family. I need to have the energy for that.”* (P6, MP).

Especially MP women described the importance of acknowledging the disorder, and the need for adjustments. They had accepted the need for medications, and not being able to breastfeed the baby. Some MP women referred to adopting another way of thinking compared to their first pregnancy. They underlined the importance of acknowledging that it is a vulnerable phase of life and that they have to take the illness seriously. Furthermore, they talked about the importance of recognising early signs of a possible postpartum relapse, and of not hesitating to ask for help from family and health services.

## Discussion

The present study addresses the knowledge gap on how perinatal women with BD relate to the increased risk for illness relapse postpartum. We wanted to learn about the women’s concerns and preparations for the dual demands of illness and motherhood. Further, we explored variations in experiences and viewpoints between subgroups, including PP and MP women. Our overarching aim was to gain knowledge that may inform prevention planning and counselling for perinatal women with BD.

### Levels of concern for illness relapse, and associations with resources and preparations

A main finding was that even if women with BD know about the postpartum risk, their level of personal concern differ. Three levels of concerns were identified, which were divided into three sub-groups, low (LC), moderate (MC) and high (HC). Group belonging was associated with different levels of perinatal deliberations and preparations.

The least deliberations and preparations were done by women in the LC group, which seems little adaptive in relation to BD postpartum risk (Jones and Craddock 2005). Thus, this group of BD women is important to identify. Denial of thoughts about risk, minimisation and indifference are reasonable indicators of a low level of concern.

Some characteristics of our LC group may be helpful in identifying and understanding a low concern stance. All the women in the LC group, had a current perception of wellness with regard to BD. Indeed, a recurrent disorder such as BD can confound risk awareness. Pregnant women who are euthymic may believe this to last, although euthymia during pregnancy is not protective of postpartum illness relapse (Doyle et al. 2012). Still, the mere presence of euthymia in pregnancy seems to be a too simplistic explanation for low concern, since other euthymic women in our sample were more concerned of postpartum illness relapse.

Noteworthy, the proportion of unplanned pregnancies was markedly high in the LC group. The overall decision making process of whether to continue the pregnancy may have overshadowed a specific focus on postpartum risk. This is worth keeping in mind, since unplanned pregnancies are found to be more common among women with BD than among healthy women, especially for primiparous women (Marengo et al. 2015).

Additionally, more complicated psychological conflicts may interfere with the process of acknowledging personal postpartum risks. The observation of longlasting relational conflicts for all women in the LC group is notable. Some women’s narratives were characterised by unresolved relational traumas. This may imply that more profound defensive processes are at play in certain cases of low concern perception.

Finally, the majority of the youngest women in our sample were in the LC group. They were almost exclusively PP. A large body of literature documents more challenges in adapting to the responsibilities of motherhood among young mothers, than in older mothers (Wakschlag and Hans 2000). For some young women, acknowledging the additional postpartum challenges may be too demanding to incorporate in their maternal transition process, thus leading to minimisation. Within a framework of additional risk factors, such as lower level of education and work participation, these young PP women represent a particularly vulnerable subgroup among women with low level of concern.

A sufficient amount of resources and preparations was found among women with moderate concern for illness relapse. Throughout the interviews, the women balanced a personal recognition of the risk with descriptions of resources and preparations. Women belonging to the MC group were characterised by having higher education and work participation, more planned pregnancies, and a longer period with mood stability. Altogether, the MC group was characterised by little additional risk, beyond the BD postpartum risk per se.

The HC group was more diverse with regard to level of resources and preparations, as well as sociodemographic factors, than the LC and MC groups. However, the women in the HC group shared clinical characteristics such as mood instability before and during pregnancy, and having had prior severe illness episodes and multiple hospitalisations. Their narratives were characterised by emotional activation and recurrent and elaborate descriptions of illness episodes. Together, this makes their high level of concern understandable.

Within the HC group, approximately half of the women had made the most comprehensive preparations of the whole sample, and mobilised extensive supportive resources. Hence, they had managed to transform their high level of concern into adequate behaviours. Their examples of goal-directed preparations challenge a stigmatising view of women with BD as poorly coping (Dolman et al. 2016; Wittkowski et al. 2014).

The remaining women in the HC group, all PP, had limited resources and preparations. We argue that these women also represent a particularly vulnerable subgroup that is critical to identify and offer comprehensive follow-up. Also, a persistent high level of concern can generate excessive negative stress for both mother and foetus (Glover 2011).

### Content of perinatal concerns, resources and preparations

Overall, PP and MP women had the same types of perinatal concerns, and envisioned similar resources and preparations. They differed on two subthemes: “The unknown”, a concern solely expressed by PP women, and “Mood episodes affecting foetus or infant”, where the MP women mostly focused toddlers or older children.

For both groups, the most significant concerns were the impact of mood episodes on mothering and on the partner.

Concerns for mothering are recurrent in studies on women with severe mental illness, including reports of a pervasive fear of being a bad parent and self-stigma (Diaz-Caneja and Johnson 2004; Dolman et al. 2013; Dolman et al. 2016). The women in our sample communicated a more conditioned view—mothering is affected only in case of active mood episodes. This highlights that women with severe mental illness may have varying views on own mothering, where nuances are important to recognise. The conditioned view in our sample may in part be associated with many women having managed relatively well in different areas of life (see Table 1). Indeed, life context for women with severe mental illness (education, employment, partner, housing etc.) has been found to be associated with their mothering (Bybee et al. 2003; Oyserman et al. 2000). Furthermore, reduced self-stigma among individuals with BD has been associated with education above primary school and employment (Brohan et al. 2011).

The women’s concerns for their partners’ demanding position, match partners’ descriptions of living with a spouse with BD (Granek et al. 2016; Tranvåg and Kristoffersen 2008). In addition, the “ordinary” burden is likely to increase in the postpartum periode, as the woman will have extensive needs for support to prevent illness relapse. Another critical matter is to secure the care needs of the infant in case of mood episodes. A trust in the father taking over the caregiving during illness relapse, may partly explain that only two women in our sample mentioned fear of child protection services. Some women provided particularly good descriptions of well functioning relationships, where the couple work as a team with regard to the BD. This is positive, since a good partner relationship has been found protective against emotional stress in the perinatal period (Røsand et al. 2011; Røsand et al. 2012).

On the other hand, our findings underline that partner issues may increase postpartum vulnerability. The partner may not have the capacity to give the woman support, either because he becomes too affected by her mood episodes, or has own mental/medical health problems. His problems may even be the main priority for the couple.

Moreover, if a partner is naïve to mood episodes, it is more difficult for him to take the illness into account, recognise early signs of mood deviations and to cope with a possible severe postpartum illness episode, such as a psychosis. These outlined partner matters are critical to address in perinatal counselling. If challenges are evident, the couple needs assistance in finding adaptive strategies.

Concerns evenly relevant for PP and MP women, were postpartum depression, negative medication effects on the child, and heredity of BD. The concerns for depression correspond with this being the most commonly reported postpartum episode polarity for women with BD (Driscoll et al. 2017; Freeman et al. 2002; Viguera et al. 2011). Concerns for medication and heredity has similarly been reported by BD women in the preconception phase (Dolman et al. 2016; Stevens et al. 2017), confirming them as central perinatal themes. In the present study, these were highlighted in the women’s request for updated and qualified guidance from specialist mental health services.

The fear of postpartum psychosis was related to diagnosis with BD I, not parity. Psychological after-effects of postpartum psychosis are found to last for years (Robertson and Lyons 2003). Our findings indicate that psychotic episodes outside the postpartum period are also affecting the women’s perinatal concerns.

The emphasis on family support correspond with BD women’s statements of what is important in their decision making about pregnancy (Dolman et al. 2016; Stevens et al. 2017). Family conflicts and geographical distances may complicate support. It is also a challenge that traditional family network support has been reduced in Western culture, leaving the woman and the partner more alone in providing each other support (Eberhard-Gran et al. 2010). It is valuable to consider and concretise the realistic extent of family support for each couple.

Psychological theories of the perinatal period highlight mental processes, where self-representations need to be reorganised to encompass new roles (Cohen and Slade 2000; Stern 1995). We argue that an enduring mental illness as BD demands a crucial reorganisation task in balancing and integrating the two identities of being a “patient with BD” and a becoming mother. Low risk recognition, or a self-stigmatising position (Dolman et al. 2013), may both be viewed as tilting out of this balance. In our view, the daily life adjustments and mental strategies described in our sample correspond to this reorganisation process. However, it is notable that some MP women mainly recognised this process in their current pregnancy. It evolved after “lessons learned” by not taking the “patient with BD” sufficiently into account when becoming mothers the first time. This seems important to keep in mind in counselling with PP women.

## Clinical implications

Based on our findings, we suggest a stepwise approach in prevention planning and perinatal counselling. The first step includes an evaluation of whether the woman is aware of the BD postpartum risk for illness relapse, and how she relates to the risk. A relevant question is whether her subjective risk perception corresponds with an objective risk status. In case of a low (i.e. minimisation of the risk) or high level of concern, possible reasons need to be identified in a dialogue.

Low concern may be associated with euthymia in pregnancy, insufficient knowledge of particular risk factors, unplanned pregnancy, or more complicated psychological conflicts in acknowledging own risk. In order to relate to the risk more personally and in-depth, women with low concern may need to have more proactive counselling, where their individual risk factors are discussed. However, this need may be ignored by health professionals because of the undemanding presence of these women, compared to women with high concern and anxiety. To prevent this from happening, health professionals need to be extra attentive and explorative when encountering LC women.

A high level of concern may be associated with longlasting or current mood instability, prior severe illness relapses and hospitalisations. Women with high level of concern may need a particularly close and attuned counselling. Their specific concerns need to be identified. Goal-directed interventions to alleviate their stress are central. Extra attention should be given to women with a high level of concern with little resources and preparations. In general, PP women are more vulnerable than MP women.

Further, we propose that the perinatal concerns identified in the present study are relevant issues to address in counselling, regardless of women’s levels of concern. However, it is imperative to address them in ways that do not increase the women’s concerns. The aim is to give the women opportunities to share their thoughts and deliberations, and encourage and support them in making adaptive preparations. This includes supporting the women in mobilising supportive relationships, adjusting daily life, and heightening their awareness of useful mental strategies.

If the woman has a cohabitating partner, it is clearly beneficial if (s)he is included in the prevention planning. Women who choose to be medication-free in pregnancy and postpartum may need extra counselling on how to prepare for, prevent, and manage illness relapse.

## Strengths and limitations

The study has its strength in contributing rich and multiple descriptions of perinatal concerns and preparations in women with BD. The qualitative exploratory nature of the study gave women opportunity to describe their experiences and views in detail.

However, there are limitations to point out. We do not know the characteristics of the women who declined to participate when informed by collaborating health workers. Our sample may represent a skewed “resourcefulness” in having the capacity to participate in a longitudinal research project in a demanding and sensitive phase of life. Therefore, the participants may have had fewer concerns and more preparations to report than those not participating. An important factor that limits the generalisability of the findings is that perinatal women without partners are not represented. Their concerns and preparations are likely to differ.

Seven women were interviewed postpartum. Their recall might have been biased because of not remembering their concerns in pregnancy or having revised their viewpoints after birth. Still, it is our evaluation that these women gave rich and nuanced descriptions of their pregnancy.

Not all women were euthymic when interviewed. Four were affected by mild depressive symptoms, and one woman was mildly hypomanic. This may have biased their descriptions and reflections. On the other hand, shifting moods represent the nature of living with BD.

An important remark concerns the result section. We use quantification of the qualitative data to report accurate occurrences of viewpoints. This makes the basis for our interpretative claims more transparent. However, the reported views are spontaneously expressed in the interviews. We do not know the “true” number of women who actually held certain views, since some may have had similar views without expressing them. Thus, the proportions reported may be conservative.

## Conclusion

The findings from the present study highlight the importance of including a psychological and psychosocial focus in perinatal prevention planning and counselling. Even if women with BD are informed about the increased risk of illness relapse postpartum, they relate to it differently. Their level of personal concern impacts their perinatal deliberations and preparations, which in turn may impact postpartum adjustment. When counselling these women, it is important to assess their personal risk recognition, perinatal concerns and available resources and preparations, and support them accordingly. Extra attention should be given to women with a low level of concern, and women with a high level of concern who have limited resources and preparations. These women represent particularly vulnerable subgroups that are critical to identify and offer comprehensive follow-up. Primiparity increases the vulnerability even more.

This article contributes to an emerging clinical knowledge base for perinatal women with BD, in which disorder-centered and patient-centered perspectives are integrated and complement each other.

## Abbreviations

BD: bipolar disorder
PP: primiparous
MP: multiparous
LC: low level of concern
MC: moderate level of concern
HC: high level of concern
